# Experiment Study for Wrist-Wearable Electro-Tactile Display

**DOI:** 10.3390/s21041332

**Published:** 2021-02-13

**Authors:** Xiong Lu, Minxu Lin, Shouchun Wang, Xusheng Hu, Hongbin Yin, Yuxing Yan

**Affiliations:** College of Automation Engineering, Nanjing University of Aeronautics and Astronautics, Nanjing 211106, China; lmx@nuaa.edu.cn (M.L.); m15850722130@163.com (S.W.); hxs19920108@163.com (X.H.); shyinhongbin@nuaa.edu.cn (H.Y.); yanyuxing@nuaa.edu.cn (Y.Y.)

**Keywords:** electro-tactile display, wrist-wearable, electrode array, recognition rate

## Abstract

Tactile sensation is a promising information display channel for human beings that involves supplementing or replacing degraded visual or auditory channels. In this paper, a wrist-wearable tactile rendering system based on electro-tactile stimulation is designed for information expression, where a square array with 8 × 8 spherical electrodes is used as the touch panel. To verify and improve this touch-based information display method, the optimal mode for stimulus signals was firstly investigated through comparison experiments, which show that sequential stimuli with consecutive-electrode-in-active mode have a better performance than those with single-electrode-in-active mode. Then, simple Chinese and English characters and 26 English characters’ recognition experiments were carried out and the proposed method was verified with an average recognition rate of 95% and 82%, respectively. This wrist-wearable tactile display system would be a new and promising medium for communication and could be of great value for visually impaired people.

## 1. Introduction

Tactile rendering provides users with a bidirectional communication channel of rich information, which is superior to the traditional two main single-channel human–computer interfaces: visual and auditory rendering [1]. Tactile rendering can enhance the realism and immersion of a virtual reality system with a specific human–computer interface device. Several types of tactile displays have been developed, of which electrostatic-type tactile displays have attracted widespread attention.

Peruzzini et al. [2] combined electro-tactile and mechanical vibration and used a tactile pad which is composed of 256 pin electrodes to distinguish different virtual materials as well as to identify the material class.

Kajimoto et al. [3] proposed a real-time pulse width modulation method. By measuring the relationship between skin resistance and absolute threshold, a function is found to determine the suitable pulse width from skin resistance. The method can stabilize the perception of electro-tactile based on the variability of traditional perception. The results show that the proposed method suppresses spatial variation and reduces temporal change.

Shen et al. [4] presented a new dynamic fingertip skin bioimpedance model based on previous experimental findings. This model is quite useful in developing an electro-tactile haptic rendering system that can regulate stimulation current to a reference current or adapt the stimulation current from the changing electro-bioimpedance of the fingertip skin.

Franceschi et al. [5] developed an electro-tactile stimulation system based on piezoelectric polymer sensors, which are motivated by the need for bidirectional communication between an amputee and a prosthetic limb. The study results on orientation, position and direction of single lines recognition verify the feasibility of artificial skin-electro stimulation systems for prosthetic applications.

To reduce the threshold voltage required to stimulate tactile receptors, Kitamura et al. [6] innovatively proposed a needle electrode array to carry out a threshold voltage experiment of electro-tactile stimulation. The average threshold voltage for electro-tactile stimulation in the experiment was about 10 V.

To further decrease the threshold voltage required to stimulate tactile receptors, Tezuka et al. [7] developed a new tactile display device called the “Two-Side Needle-Flat Device” based on previous research of Kitamura. In comparison with the “One-Side Needle Device” and the “Flat-electrode Device,” the proposed device not only reduces the threshold voltage but also increases the voltage range of perception.

Later, Tezuka et al. [8,9] further studied electro-tactile stimulation and developed a sheet-type electro-tactile device with microneedle electrodes to transfer information. The device is capable of increasing the variety of tactile sensations and the amount of available information with a low voltage.

In addition to touching and perceiving tactile rendering through the fingertip [10,11,12,13], the lips [14], tongue [15,16,17], forehead [18], abdomen [19], forearm [8], back [20] and residual nerves [21] also have been utilized to express information. These various tactile displays are usually obstructive because of the invasiveness of the stimulation or the large dimension being a burden to a healthy person. Furthermore, the widely used fingertip-based tactile displays are unsuitable for users whose hands are often engaged in tasks such as driving, physical exercise, instrument playing, etc. Nowadays, with more and more research on tactile rendering, various wearable devices [22] have been proposed, aiming to be more portable, skin-friendly, and easy-to-wear.

Lee et al. [23] presented the design of a wearable textile-based electro-tactile display embedded in a wristband that can provide the user with an unobtrusive alert that is easy to distinguish. However, the purpose of their system was only restricted to the aspects of directional patterns.

In the previous study of the authors [24], a wrist-wearable system based on electro-tactile stimulation with 8 × 8 spherical electrodes was proposed, which is capable of transferring information whenever the surroundings are noisy or someone cannot acquire information in time—for example, when driving a car. The study made a comparison between the static display method, which displayed all the electrodes at a time, and the dynamic display method, which displayed one electrode at a time according to some specific orders. The experimental results showed that the dynamic display method could greatly improve the success rate of recognition for the wrist.

Based on the previous study, this paper performs further experimental research on two different sequential stimulation modes for the dynamic display method, bringing a better tactile rendering performance to human operators. This paper is organized as follows. The principle of electro-tactile stimulation is explained and the hardware of the electro-tactile rendering system is designed and implemented in Section 2. In Section 3, the experimental study is carried out, including optimal parameters for the stimulating signal experiment and the character recognition experiment. Finally, the conclusion of this paper is stated in Section 4.

## 2. System Structure

**Figure 1:**
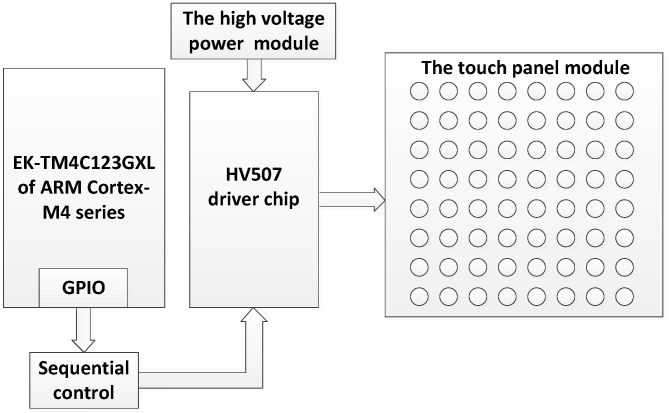
The schematic diagram of the electro-tactile display system.

**Figure 2:**
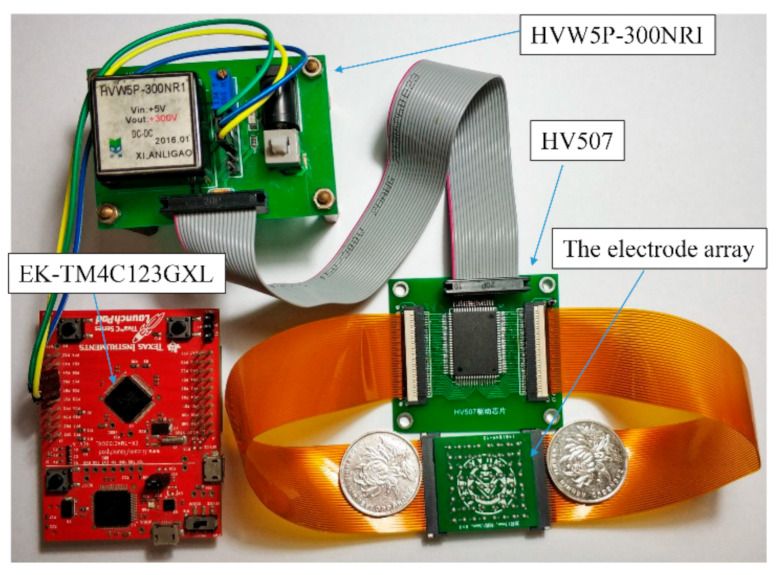
Actual structure of the proposed electro-tactile display system.

A wrist-wearable tactile rendering system based on electro-tactile stimulation is designed and implemented. Figure 1 and Figure 2 show the schematic diagram and the actual structure of the proposed system, respectively.

**Figure 3:**
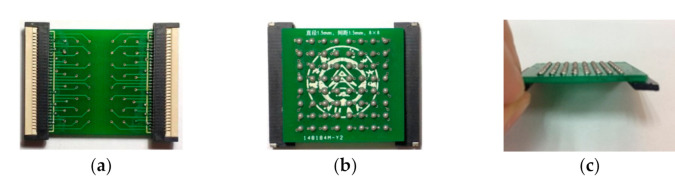
The wrist-wearable touch panel module. (**a**) Top view; (**b**) bottom view; (**c**) side view.

The proposed system consists of four main modules: (1) The touch panel module is a square array composed of 8 × 8 spherical electrodes, where the diameter of each electrode is 1.5 mm and the center distance between adjacent electrodes is 3 mm. Each spherical electrode was fabricated with solder tin melted on printed circuit board (PCB) pads designed by the authors. The area of the 8 × 8 spherical electrode array is 27 × 27 mm and is similar to a coin in size, as shown in Figure 2 and Figure 3. The touch panel module is the human-computer interaction interface, and this module is connected to the stimulating signal driver module using a flexible flat cable (FFC). (2) The central control module is constructed with an EK-TM4C123GXL board (ARM Cortex-M4 microcontroller), which is responsible for controlling the driving module of the electrode array. (3) The stimulating signal driver module is implemented with an HV507 chip, which is a low-voltage serial to high-voltage parallel converter. It is utilized to output a high voltage on selected electrodes of the electrode array with designed output sequences. (4) The high-voltage power module (HVW5P-300NRI from Xi’an Ligao Elec. Tech. China) provides the high voltage (adjustable from 0 to 300 V) for the stimulating signal driver module.

## 3. Experiments

To study tactile perception characteristics of people’s wrists, experiments to find optimal parameter values of the stimulating signal and for characters’ recognition, including simple Chinese and English characters’ recognition and 26 English characters’ recognition, were carried out.

Five subjects including four males and one female were invited to participate in the experiments. The five subjects received a thorough explanation of the experimental methods. Each subject wiped his/her wrist by using alcohol before the experiments to remove sweat and grease, which had a great influence on the experiments.

The threshold voltage was the minimum voltage at which the subjects begin to feel the tactile sensation. The threshold voltage experiments in our previous research [24] also proved that the threshold voltage was the lowest when the frequency of the stimulation signal was 100 Hz with a duty cycle of 35%. Besides, a one-hour learning session was included in these experiments to familiarize the subjects with the feeling of electro-tactile stimulation.

### 3.1. Optimal Parameters for the Simulating Signal for Electro-Tactile Rendering

#### 3.1.1. Two Electro-Tactile Rendering Modes

**Figure 4:**
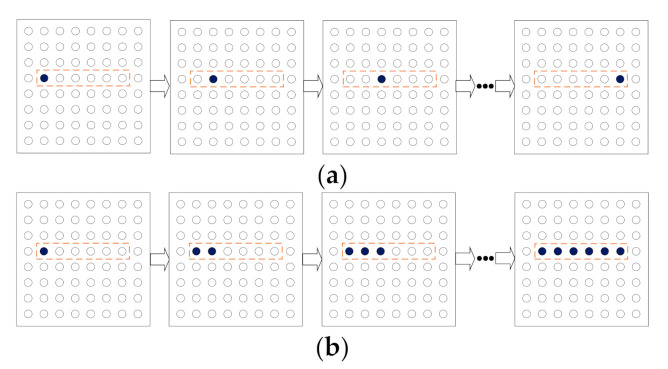
The process of rendering the symbol “一”: (**a**) the sequential stimulating signal with single-electrode-in-active (SSS) mode; (**b**) the sequential stimulating signal with consecutive-electrode-in-active (SSC) mode.

Two different sequential stimulating modes for the electro-tactile electrode array have been studied in this paper, i.e., the sequential stimulating signal with single-electrode-in-active (SSS) mode and the sequential stimulating signal with consecutive-electrode-in-active (SSC) mode. The electrodes corresponding to the pixels for rendering the symbol “一”, which is identical to the Chinese character “one”, were stimulated successively according to the stroke order. In the SSS stimulating mode, when the stimulating signal is outputted for the next electrode, the signal for the previous electrode will be turned off. Therefore, the stimulating signal was outputted to one single electrode in the whole procedure of symbol rendering, as presented in Figure 4a. However, in the SSC mode, when the stimulating signal is outputted to the next electrode, signals for the previous electrodes will remain available, which is shown in Figure 4b.

#### 3.1.2. Procedure

Optimal parameters for the stimulating signal include optimal step duration (SD) and optimal step-pause duration (SPD). During the procedure of character rendering, the SD means the time duration of the “ON” state of an electrode, i.e., the time duration for outputting the high-voltage stimulating signal. The SPD refers to the time interval between the “ON” states for two adjacent electrodes. Both of them are believed to have a great impact on the performance of tactile rendering. The optimal frequency (100 Hz) and duty cycle (35%) values obtained from the threshold voltage experiment in our previous research [24] are used in the following experiments.

In this experiment, each of the five subjects were asked to evaluate the score of tactile perception of the proposed tactile display system on his or her wrist, according to the following five-level criteria.

We randomly chose one column of the electrodes on the touch panel and applied voltage signals with the SSS mode and the SSC mode respectively during this experiment, and the tactile perception for electro-tactile rendering was divided into five levels (grades 1–5). The first level (grade 1) indicated that subjects could not feel any movement of electrodes. The second level (grade 2) indicated that subjects could feel a few electrodes move in turn. The third level (grade 3) indicated that about half of the electrodes could be felt. The fourth level (grade 4) indicated that subjects could feel most of the electrodes. The fifth level (grade 5) indicated that subjects could clearly feel every electrode.

#### 3.1.3. Result and Discussion

The experiment results for these two stimulating modes, i.e., the tactile perception scores, are illustrated in Table 1 and Table 2, respectively. It is shown that the shorter the SPD, the lower the perception score would be. This was due to the shorter interval time resulting in a faster transfer speed between adjacent electrodes. Therefore, subjects could hardly distinguish the tactile stimulation of adjacent electrodes in that case. It could be seen that the optimal SD was 0.1 s (10 periods), and the optimal SPD was 1 s, where each subject could distinguish each electrode clearly with the optimal SD and SPD parameters. The SSC mode worked better than the SSS mode when the SPD was large enough (for example, SPD = 1.0 s), i.e., the stimulating signal with SSC mode provides higher tactile perception scores. We believe that the better performance of the SSC mode is attributed to more activated electrodes and, subsequently, more employed current in the system, and the remaining information for intended tactile rendering patterns.

### 3.2. Character Recognition Experiments

To research the tactile perception characteristics of people’s wrists with characters, simple Chinese and English characters’ recognition experiments and 26 English characters’ recognition experiments were conducted. The optimal SD value (SD = 0.1 s) and the optimal SPD value (SPD = 1.0 s) according to the above experiment were utilized with the SSC mode. Each subject also chose an appropriate voltage value before each experiment and perceived it 20 times for each lattice model character.

#### 3.2.1. Simple Chinese and English Characters Recognition

**Figure 5:**
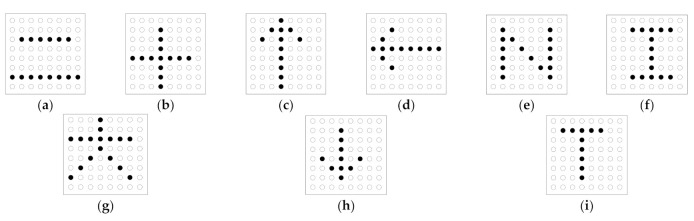
Simple characters’ lattice models: (**a**) “二”; (**b**) “十”; (**c**) “↑”; (**d**) “←”; (**e**) “N”; (**f**) “I”; (**g**) “大”; (**h**) “↓”; (**i**) “T”.

As shown in Figure 5, three groups of simple Chinese and English characters with three characters in each group were designed for this experiment. We randomly selected one of the three groups which included Chinese characters (“二”, “十”, and “大”) (Figure 5a,b,g), arrows (“↑”, “←”, and “↓”) (Figure 5c,d,h), and English characters (“N”, “I”, and “T”) (Figure 5c,d,h). The nine designed simple characters were shown to each participant visually before the experiment. However, we deliberately chose one character from the first two characters in each group in the experiment, which was not conveyed to the participants. Therefore, only six designed characters (Figure 5a–f) were rendered in the experiment.

Table 3 shows the success rate of simple Chinese and English character recognition. It is seen that the average successful recognition rate of the simple Chinese and English characters was approximately 95%, which demonstrates the validity of the proposed system. The SSC mode adopted in this experiment greatly improved the success rate of each character. During the experiment, it was found that the characters with similar strokes are easily confused, resulting in recognition errors.

#### 3.2.2. 26 English Characters’ Recognition

**Figure 6:**
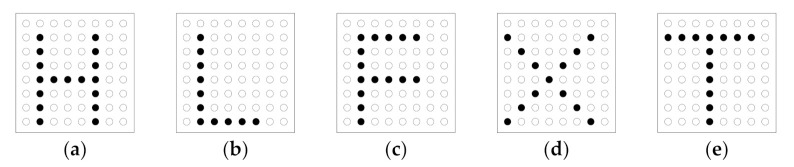
The lattice model of selected five English characters: (**a**) “H”; (**b**) “L”; (**c**) “F”; (**d**) “X”; (**e**) “T”.

In this experiment, we increased the difficulty of the character recognition experiment. We randomly chose five English characters from all 26 capitalized English characters for the experiment, as shown in Figure 6. The designed patterns of the 26 capital English characters were not illustrated to the five subjects in any form. The subjects did not know the selected five characters before the experiment, so they needed to give their answers from 26 English characters.

Table 4 shows the success rate of recognizing 26 English characters. The average success rate of 26 English character recognition was approximately 82%. It had a certain degree of decline compared to that of the simple Chinese and English characters recognition experiment. The decline was understandable owing to the abundance of the word library, and this result further demonstrated the validity of the proposed system.

Additionally, similar characters were also easily confused in this experiment, such as “I” and “T”, “E” and “F”, “H” and “N”, and “X” and “Y”.

## 4. Conclusions

This paper presents a wrist-wearable tactile rendering system based on electro-tactile principles. A series of experiments including optimal parameter values of the stimulating signal experiment and character recognition experiments including simple Chinese and English characters’ recognition and 26 English characters’ recognition were carried out to explore the possibility of using the proposed system as a tool for transferring information to wrists. This proposed system achieved high performance owing to adapting the SSC mode, which improves the average success rate of character recognition. Besides, this proposed system is lightweight, compact, and portable.

Portable and refreshable character expression remains a great challenge in tactile rendering. Compared to previous character recognition systems, such as that having a recognition success rate of 76.2% for large (52 mm) and 56.4% for small (20 mm) English characters with an electro-tactile display of 63 (7 × 9) electrodes with 2-mm center intervals using a single finger [25], and that with a success rate of 71.9% for lower-case English characters (14 mm high, one letter per second) and 91.3% after a 5-min training period with a stylus-writing-on-hand system [26], the average success rate of simple Chinese and English characters and 26 English characters’ recognition was about 95% and 82%, respectively, for the proposed tactile display system. These experimental results verify the effectiveness of the proposed system. The proposed system can contribute to the further development of information transfer systems that use tactile sensation. It can be used to express information portably in various applications, such as tasks in degraded visual environments, scenarios where people’s hands are engaged in tasks ( like driving and exercising), and daily tasks for both healthy and visually impaired people.

The number of participants in our research was limited. In the future, more experiments with a larger number of participants can be carried out to further study the performance differences between Chinese character recognition and English character recognition, to find effective modeling methods for rendering complex Chinese characters, and to design methods for rendering non-textual tactile graphics. Moreover, tactile displays combining the electro-tactile principle and other tactile display methods such as electrostatic tactile principle may be promising for further improving the success rate for recognizing text and graphics information.

## Figures and Tables

**Table 1:** Average perception level of electro-tactile stimulation with the SSS mode.

	SD = 0.25 s	SD = 0.20 s	SD = 0.15 s	SD = 0.10 s	SD = 0.05 s
SPD = 1.0 s	4.0	3.8	4.6	4.6	3.2
SPD = 0.8 s	3.4	3.4	3.8	4.0	3.2
SPD = 0.6 s	3.6	3.8	3.2	3.4	3.2

**Table 2:** Average perception level of electro-tactile stimulation with the SSC mode.

	SD = 0.25 s	SD = 0.20 s	SD = 0.15 s	SD = 0.10 s	SD = 0.05 s
SPD = 1.0 s	4.8	4.6	4.8	5.0	3.4
SPD = 0.8 s	4.2	4.2	4.4	4.2	3.2
SPD = 0.6 s	3.0	3.0	3.0	3.0	2.0

**Table 3:** Successful rate of the simple Chinese & English characters recognition.

Subject	“二”	“十”	“↑”	“←”	“N”	“I”
S1	95%	95%	100%	100%	100%	100%
S2	100%	100%	90%	95%	95%	95%
S3	100%	95%	90%	90%	90%	90%
S4	100%	95%	95%	90%	95%	95%
S5	100%	95%	100%	90%	90%	90%
Average	99.0%	96.0%	95.0%	93.0%	94.0%	94.0%

**Table 4:** Success rate of 26 English characters’ recognition.

Subject	“H”	“L”	“F”	“X”	“T”
S1	85%	85%	80%	85%	85%
S2	90%	85%	80%	90%	90%
S3	80%	85%	80%	75%	80%
S4	80%	80%	75%	80%	85%
S5	80%	80%	75%	80%	80%
Average	83.0%	83.0%	78.0%	82.0%	84.0%

## Data Availability

The data presented in this study are available on request from the corresponding author.

## References

[1] Salisbury K., Conti F., Barbagli F. (2004). Haptic rendering: Introductory concepts. IEEE Eng. Med. Boil. Mag..

[2] Peruzzini M., Mengoni M., Germani M. Virtual Tactile Simulation: A Novel Display and Effects on Users’ Perception. Proceedings of the Asme/iscie 2012 International Symposium on Flexible Automation.

[3] Kajimoto H. (2011). Electrotactile Display with Real-Time Impedance Feedback Using Pulse Width Modulation. IEEE Trans. Haptics.

[4] Shen Y., Gregory J., Xi N. (2014). Stimulation current control for load-aware electrotactile haptic rendering: Modeling and simulation. Robot. Auton. Syst..

[5] Franceschi M., Seminara L., Pinna L., Dosen S., Farina D., Valle M. Preliminary evaluation of the tactile feedback system based on artificial skin and electrotactile stimulation. Proceedings of the 2015 37th Annual International Conference of the IEEE Engineering in Medicine and Biology Society (EMBC).

[6] Kitamura N., Chim J., Miki N. (2015). Electrotactile display using microfabricated micro-needle array. J. Micromech. Microeng..

[7] Tezuka M., Kitamura N., Tanaka K., Miki N. (2016). Presentation of Various Tactile Sensations Using Micro-Needle Electrotactile Display. PLoS ONE.

[8] Tezuka M., Ishimaru K., Miki N. (2017). Electrotactile Display Composed ofTwo-dimensionally and Densely Distributed Microneedle Electrodes. Sens. Actuators A Phys..

[9] Tezuka M., Kitamura N., Miki N. (2016). Information transfer using wearable thin electrotactile displays with microneedle electrodes. Jpn. J. Appl. Phys..

[10] Kaczmarek K.A., Tyler M.E., Bach-Y-Rita P. Electrotactile haptic display on the fingertips: Preliminary results. Proceedings of the 16th Annual International Conference of the IEEE Engineering in Medicine and Biology Society.

[11] Gregory J., Xi N., Shen Y. Towards on-line fingertip bio-impedance identification for enhancement of electro-tactile rendering. Proceedings of the 2009 IEEE/RSJ International Conference on Intelligent Robots and Systems.

[12] Shin H., Watkins Z., Huang H., Zhu Y., Hu X. (2018). Evoked haptic sensations in the hand via non-invasive proximal nerve stimulation. J. Neural Eng..

[13] Arakeri T.J., Hasse B.A., Fuglevand A.J. (2018). Object discrimination using electrotactile feedback. J. Neural Eng..

[14] Shim J., Liu W., Tang H. (2006). System development for multichannel electrotactile stimulation on the lips. Med Eng. Phys..

[15] Bach-Y-Rita P., Kaczmarek K.A., Tyler M.E., Garcia-Lara J. (1998). Form perception with a 49-point electrotactile stimulus array on the tongue: A technical note. J. Rehabil. Res. Dev..

[16] Bach-Y-Rita P., Tyler M.E., Kaczmarek K.A. (2003). Seeing with the Brain. Int. J. Hum. Comput. Interact..

[17] Jeffs A., Warwick K. Sensory Perception through an Electro-tactile Stimulus Array on the Tongue. Proceedings of the 2013 IEEE International Conference on Systems, Man, and Cybernetics.

[18] Kajimoto H., Kanno Y., Tachi S. Forehead Electro-Tactile Display for Vision Substitution. Proceedings of the EuroHaptics.

[19] Kaczmarek K. (2000). Electrotactile adaptation on the abdomen: Preliminary results. IEEE Trans. Rehabil. Eng..

[20] Seps M., Dermitzakis K., Hernandez-Arieta A. Study on lower back electrotactile stimulation characteristics for prosthetic sensory feedback. Proceedings of the 2011 IEEE/RSJ International Conference on Intelligent Robots and Systems.

[21] Christie B.P., Charkhkar H., Shell C.E., Marasco P.D., Tyler D.J., Triolo R.J. (2019). Visual inputs and postural manipulations affect the location of somatosensory percepts elicited by electrical stimulation. Sci. Rep..

[22] Gallo S., Son C., Lee H.J., Bleuler H., Cho I.-J. (2015). A flexible multimodal tactile display for delivering shape and material information. Sensors Actuators A: Phys..

[23] Lee S.C., Starner T. Stop burdening your eyes: A wearable electro-tactile display. Proceedings of the 2008 12th IEEE International Symposium on Wearable Computers.

[24] Hu X., Lu X., Sun H. (2017). The Wearable Tactile Information Expression System Based on Electrotactile Rendering. Transactions on Edutainment XIII.

[25] Uematsu H., Suzuki M., Kanno Y., Kajimoto H. Tactile Vision Substitution with Tablet and Electro-Tactile Display. Proceedings of the International Conference on Human Haptic Sensing and Touch Enabled Computer Applications.

[26] Hasegawa K., Sakurai T., Makino Y., Shinoda H. (2016). Character Reading via Stylus Reproducing Normal Handwriting Motion. IEEE Trans. Haptics.

